# Guidelines for Discontinuation of Antipsychotics in Patients Who Recover From First-Episode Schizophrenia Spectrum Disorders: Derived From the Aggregated Opinions of Asian Network of Early Psychosis Experts and Literature Review

**DOI:** 10.1093/ijnp/pyac002

**Published:** 2022-04-22

**Authors:** Asian Network of Early Psychosis Writing Group, Christy L M Hui, Eric Y H Chen, Verma Swapna, Hiromi Tagata, Masafumi Mizuno, Chen‑Chung Liu, Hiroyoshi Takeuchi, Sung-Wan Kim, Young-Chul Chung

**Affiliations:** Department of Psychiatry Unive, University of Hong Kong, Hong Kong, SAR, China; Department of Psychiatry Unive, University of Hong Kong, Hong Kong, SAR, China; State Key Laboratory of Brain and Cognitive Sciences, University of Hong Kong, Hong Kong, SAR, China; Institute of Mental Health, Singapore; Duke-NUS Medical School, Singapore; Department of Neuropsychiatry, Toho University School of Medicine, Tokyo, Japan; Department of Neuropsychiatry, Toho University School of Medicine, Tokyo, Japan; Tokyo Metropolitan Matsuzawa Hospital, Tokyo, Japan; Department of Psychiatry, National Taiwan University Hospital, Taipei, Taiwan; Department of Psychiatry, College of Medicine, National Taiwan University, Taipei, Taiwan; Department of Neuropsychiatry, Keio University School of Medicine, Tokyo, Japan; Schizophrenia Program, Centre for Addiction and Mental Health, Toronto, ON, Canada; Mindlink, Gwangju Bukgu Mental Health Center, Gwangju, Korea; Department of Psychiatry, Chonnam National University Medical School, Gwangju, Korea; Department of Psychiatry, Jeonbuk National University Medical School, Jeonju, Korea; Research Institute of Clinical Medicine of Jeonbuk National University – Biomedical Research Institute of Jeonbuk National University Hospital, Jeonju, Korea

**Keywords:** Antipsychotics, discontinuation, first episode, GRADE, non-affective psychosis

## Abstract

**Objective:**

Antipsychotic discontinuation has been a long-standing clinical and medicolegal issue. The Asian Network of Early Psychosis developed guidelines for antipsychotic discontinuation in patients who recover from first-episode non-affective psychosis. We reviewed the existing studies and guidelines on antipsychotic discontinuation to develop guidelines for antipsychotic discontinuation in such patients.

**Methods:**

We reviewed the relevant studies, reviews, guidelines, and ongoing trials related to antipsychotic discontinuation in patients with first-episode psychosis or schizophrenia. The quality of randomized controlled trials was assessed using the Grading of Recommendations Assessment, Development, and Evaluation approach.

**Results:**

Most studies had low to very low quality, and 2 had moderate quality. All studies, except 1, advised against antipsychotic discontinuation because of higher relapse rates in the antipsychotic discontinuation group (19%–82% at 1-year follow-up) than the treatment maintenance group compared with the maintenance group. Based on expert opinion and Grading of Recommendations Assessment, Development, and Evaluation evidence of trials, guidelines have been recommended for future discontinuation studies on patients with first-episode schizophrenia spectrum disorders.

**Conclusions:**

Currently, there are no recommendations for antipsychotic discontinuation in patients with first-episode schizophrenia spectrum disorders. However, there is a pressing need to conduct more rigorous research in remitted patients using more stringent criteria of full recovery, which can form the basis of guidelines on when and how antipsychotics should be tapered and discontinued. Studies that evaluate the patient characteristics and biomarkers that predict successful antipsychotic discontinuation are also needed.

**Significance Statement**

For patients with first-episode schizophrenia (FES), 6 of the 11 current guidelines partially recommended antipsychotic discontinuation after 1 to 2 years of treatment. However, they did not specify the details about when and how this could be attempted. We reviewed the literature, guidelines, and ongoing studies of antipsychotic discontinuation. Based on this evidence and expert opinions, we developed guidelines for antipsychotic discontinuation and optimization of patient outcomes. The 2 key points are that based on the GRADE evidence of trials, discontinuation is not recommended in patients with FES and more strict criteria for discontinuation trials is required in future research.

## Introduction

Psychotic disorders are one of the most disabling groups of severe mental disorders worldwide (Global Burden of Disease Study, 2013). They are associated not only with personal disability and suffering but also immense family burden and increased risk for suicide, physical morbidity, and premature death (Palmer et al., 2005; Saha et al., 2007; Walker et al., 2015). Antipsychotics are the mainstay in the treatment of psychosis, and they alleviate the positive symptoms effectively (Leucht et al., 2009). Following antipsychotic use, patients often enter the maintenance phase of disease. However, despite the control of positive symptoms, psychiatrists, particularly Asian psychiatrists, advise against discontinuation (Hui et al., 2019a). This advice is based on evidence that antipsychotic use does not permanently decrease dopamine activity, and their discontinuation often leads to disease recurrence (Robinson et al., 1999).

Disease relapses can undo hard-won educational and vocational gains, increase the risk for suicide, worsen symptom control, and reduce the response to treatment (Wiersma et al., 1998; Emsley et al., 2012; Chan et al., 2018). Therefore, prevention of relapse is the most important goal in the maintenance phase of the disease; a simple strategy to prevent relapse is long-term antipsychotic use during the maintenance phase. However, the long-term or permanent intake of antipsychotics is also associated with an array of drawbacks. First, while relapse prevention is the main purpose of continued medication, there are instances where it has nonetheless resulted in a relapse (i.e., psychosis breakthrough on antipsychotic maintenance medication). Not only does this represent a major barrier to successful maintenance treatment, but it also questions the necessity of such drug regimen. Second, patients often dislike long-term antipsychotic use because of stigmatization (Sajatovic and Jenkins, 2007), adverse effects (Bebbington et al., 2009), and cost (Gibson et al., 2010). Almost 80% of schizophrenia patients discontinue their medications against medical advice (Lacro et al., 2002; Zygmunt et al., 2002; Fleischhacker et al., 2003). This is probably due to the desire of patients for the state of recovery rather than just achieving its prerequisite, that is, symptomatic remission.

According to the Psychosis Recovery Inventory, which evaluated the experiences of patients after recovery from a first-episode psychosis (FEP), some patients feel that they are not “fully recovered” because of ongoing need for medications (Chen et al., 2005). Recovery is defined as a process “in which people are able to live, work, learn, and participate fully in their communities,” allowing them “to live a fulfilling and productive life despite a disability” (Resnick et al., 2005; Bellack, 2006). Whereas current guidelines indicate how antipsychotics should be maintained for the abovementioned drawbacks of medication and conceptualization of recovery, it is equally important to devise guidelines for the discontinuation of antipsychotics. The fundamental questions of whether and when this could be attempted remains controversial.

Several studies have demonstrated the benefits and risks of antipsychotic discontinuation in patients with psychosis (Kane et al., 1982; Crow et al., 1986; Gitlin et al., 2001; Wunderink et al., 2007; Chen et al., 2010). Few studies have also been conducted for the long-term outcomes of antipsychotic discontinuation (Wunderink et al., 2013; Hui et al., 2018). Trials are currently underway in Denmark, the Netherlands, the United Kingdom, and Australia to evaluate the processes, risks, and outcomes for antipsychotic discontinuation. Asian psychiatrists were found to have very diverse views on proportions of remitted patients whom they think can stop medication completely and durations of antipsychotic maintenance following absence of psychotic symptoms and criteria for discontinuation in patients with FEP (Hui et al., 2019a). Several guidelines partially recommend antipsychotic discontinuation in patients with first-episode schizophrenia (FES) but do not specify how and when this can be done safely. To answer these important but unresolved questions, the Asian Network of Early Psychosis Writing Group developed guidelines for antipsychotic discontinuation in patients recovered from first-episode non-affective psychosis. We reviewed the literature, guidelines, and ongoing studies of antipsychotic discontinuation. Based on this evidence and expert opinions, we developed guidelines for antipsychotic discontinuation and optimization of patient outcomes.

## METHODS

In January 2020, we searched the literature for studies of antipsychotic discontinuation in patients with FEP or FES, using the following keywords: “discontinuation,” “antipsychotics,” “first-episode,” “non-affective psychosis,” “schizophrenia,” and “recovery.” We selected studies that evaluated the outcomes after discontinuation or intermittent treatment of antipsychotics. We also selected studies that reported the characteristics of patients who discontinued antipsychotics and guidelines for the treatment of psychosis or schizophrenia. Eventually, review was conducted on 11 randomized controlled trials (RCTs), 2 non-RCTs, and 3 reviews. This is not a systematic review; the literature review was conducted only to provide an overview of the current evidence and enrich the subsequent discussion on antipsychotic discontinuation.

We evaluated the quality of RCTs using the Grading of Recommendations Assessment, Development, and Evaluation (GRADE) approach (Network GI, GRADE Working Group, 2016). The quality of evidence (high, moderate, low, or very low) was based on the strengths and weaknesses of studies resulting from risk for bias, imprecision, inconsistency, indirectness of results, and publication bias. The quality of evidence also reflects our confidence in the study results to support recommendations. We rated the strengths of recommendations (strong or weak/conditional) based on the extent to which the desirable consequences outweigh the undesirable consequences of antipsychotic discontinuation. A strong recommendation was made where the desirable consequences clearly outweighed the undesirable consequences. By contrast, a weak recommendation was made where the desirable consequences likely outweighed the undesirable consequences. These recommendations were used to develop the guidelines.

## RESULTS

### Short-Term Outcomes After Antipsychotic Discontinuation

Most psychiatrists are unlikely to advise antipsychotic discontinuation following remission from FEP (Thompson et al., 2016; Hui et al., 2019a) because of a high risk for relapse (Zipursky et al., 2014). The high risk for relapse was confirmed in several studies that investigated the short-term outcomes after antipsychotic discontinuation (Kane et al., 1982; Crow et al., 1986; Gitlin et al., 2001) (Table 1).

**Table 1.** Key Findings of Studies on Antipsychotic Discontinuation

									Rate of relapse (or another primary outcome)	
Study	Study design	Diagnoses and total sample size	Remission criteria	Mean dose at baseline (CPZ mg/d)	Method of discontinuation	AP taken by maintenance group	Follow-up duration after discontinuation	Definition of relapse (or another primary outcome)	Discontinuation group	Maintenance group
RCTs										
Kane et al. (1982)	Double-blind RCT	S, psychosis NOS, other psychiatric disorders, manic disorder with schizotypal features, and major depressive disorder with schizotypal features (RDC), n = 28	Stable clinical condition on maintenance treatment for ≥4 wk, within 1 y following hospital discharge	N/A	Patients given procyclidine hydrochloride for first mo and placebo for second mo of study (n = 17)	Fluphenazine hydrochloride (5–20 mg/d) or fluphenazine decanoate (12.5–50 mg every 2 wk) (n = 11)	1 y	Substantial clinical deterioration with potential for marked social impairment	41%	0%
Crow et al. (1986)	Double-blind RCT	FEP which was not unequivocally affective (PSE), n = 120	Discharged from hospital care for ≥30 d	N/A	Cross-titration to placebo over 2 mo (n = 66)	1 of 5 neuroleptics at or above stated dose (flupenthixol 40 mg/mo, chlorpromazine 200 mg/d, haloperidol 3 mg/d, pimozide 4 mg/d, trifluoperazine 5 mg/d) (n = 54)	2 y or to end of participation (defined by occurrence of a relapse or loss to follow-up)	Readmission to psychiatric care; readmission considered necessary by clinician even if for some reason not possible; active antipsychotic medication considered necessary by clinician due to features of imminent relapse	62%	46%
McCreadie et al. (1989)	Double-blind RCT	S (PSE, Feigner, RDC), n = 15	1 y maintenance treatment without history of relapse	N/A	Placebo (method of discontinuation not specified) (n = 7)	Pimozide or flupenthixol decanoate (n = 8)	1 y	Re-hospitalization due to psychotic symptoms	Hospitalization: 57%	Hospitalization: 0%
Wunderink et al. (2007)	Open-label RCT	S, SCP, SCA, brief psychotic disorder, delusional disorder, or psychotic disorder NOS (SCAN), n = 131	One rating of ≤4 on PANSS positive subscale for ≥6 mo while on maintenance treatment (exacerbation of positive symptoms for ≤1wk allowed)	N/A	Dose reduction/discontinuation (gradual symptom-guided tapering of dosage and discontinuation if feasible) (n = 65)	Maintenance treatment with preferential prescription of low-dose second-generation APs (n = 63)	1.5 y	Clinical deterioration for ≥1 wk, having consequences (i.e., augmentation of AP dosage, hospital admission, or more frequent consultations), subsequently confirmed by rating of ≥5 on at least 1 PANSS positive subscale item	43%	21%
Chen et al. (2010)	Double-blind RCT	S, SCP, SCA, brief psychotic disorder, psychosis-NOS (SCID-IV), n = 178	Scores of ≤2~4 on P1, 2, 3, 6, G9 on PANSS for ≥8 wk, CGI-S≤2, ≥1 y maintenance treatment with no history of relapse	Discontinuation group: 125.8 (≤ 92.3) Maintenance group: 153.0 (≤ 193.9) All patients: 138.8 (SD = 149.3)	Cross-titration to placebo over 4–6 wk (n = 89)	Quetiapine 400 mg/d (n = 89)	1 y	Scores of ≥3~5 on P1, P2, P3, P6, and G9 in PANSS for ≥1 wk; scores ≥3 on CGI-S and ≥5 on CGI-I	79% (Hospitalization: 16%)	41% (Hospitalization: 6%)
Boonstra et al. (2011)	Open-label RCT	S, SCP, SCA (SCID-IV) n = 20	Scores of ≤3 on PANSS positive subscale items for ≥1 y	N/A	Gradual discontinuation over 6–12 wk (n = 11)	Continuation of AP(s) originally taken by patients (n = 9)	2 y	Score of ≥4 on any PANSS positive subscale item(s) and 20% increase in total PANSS score, or hospitalization for any psychiatric indication	At 9 mo: 82% At 24 mo: 91%	At 9 mo: 12% At 24 mo: 45%
Gaebel et al. (2011)	Open-label RCT	S, SCP (ICD-10), n = 44	Sufficiently stable, defined by having no relapse (see description) in first-year post-acute phase; no need of early intervention according to decision algorithm detailed in Fig. 2 of manuscript	N/A	Stepwise drug discontinuation and targeted intermittent treatment over max. 3 mo (n = 21)	Risperidone or haloperidol 2–8 mg (n = 23)	1 y	Increase in PANSS positive subscale score >10, CGI change score ≥6, and decrease in GAF score >20	19%	0%
Wunderink et al. (2013)	Open-label RCT	S, SCP, SCA, BPS, delusional disorder, or psychotic disorder NOS (SCAN), n = 103	One rating of ≤4 on PANSS positive subscale for ≥6 mo while on maintenance treatment (exacerbation of positive symptoms for ≤1 wk allowed)	N/A	Dose reduction/discontinuation (gradual symptom-guided tapering of dosage and discontinuation if feasible) (n = 52)	Maintenance treatment with preferential prescription of low-dose second-generation APs (n = 51)	7 y	Rate of recovery (defined by scoring ≤3 in all relevant PANSS items and functional remission for at least 6 mo)	Recovery rate: 40.4%	Recovery rate: 17.6%
Emsley et al. (2014)	Double-blind RCT	S, SCP, SCA (DSM-IV), n = 33	2–3 y maintenance treatment with no history of relapse and currently in remission according to Remission in Schizophrenia Working Group criteria	N/A	Gradual tapering and discontinuation over 6 mo (n = 12)	Combination of ω-3 PUFAs and a metabolic antioxidant, α-LA (n = 21)	2 y	25% increase in PANSS total score; deliberate self-injury; emergence of clinically significant suicidal or homicidal ideation; or violent behavior resulting in significant injury to another person or significant property damage	75%	90%
Gaebel et al. (2016)	Open-label RCT	S, SCP (ICD-10), n = not given	≥1 y maintenance treatment and sufficiently stable with no clinically relevant positive symptoms	Haloperidol equivalents for discontinuation group: 2.5 (≤1.1)	Stepwise drug discontinuation and targeted intermittent treatment over max. 3 mo (n = 19)	Risperidone or low-dose haloperidol (n = not given)	1 y	Deterioration: defined as an increase in the sum of the PANSS positive and negative scores ≥10 points or CGI change score ≥6	Deterioration: 52.6% No deterioration: 47.4%	N/A
Hui et al. (2018)	Double-blind RCT	S, SCP, SCA, brief psychotic disorder, psychosis NOS (SCID-IV), n = 178	Scores of ≤2~4 on P1, 2, 3, 6, G9 on PANSS for ≥8 wk, CGI-S≤2, ≥1 y maintenance treatment with no history of relapse	Discontinuation group: 125.9 (≤ 84.1) Maintenance group: 177.4 (≤165.9) All patients: 151.6 (≤133.6)	Cross-titration to placebo over 4–6 wk (n = 89)	Quetiapine 400 mg/d (n = 89)	10 y	Proportion of patients with good or poor long-term clinical outcomes at 10 y (defined as either presence of persistent psychotic symptoms, a requirement for clozapine treatment, or death by suicide)	Poor long-term clinical outcome at 10 y: 39%	Poor long-term clinical outcome at 10 y: 21%
Non-randomized trials										
Gitlin et al. (2001)	Non-randomized, open-label intervention	S, SCA (RDC), n = 53	≥1 y on fluphenazine decanoate treatment and absence of notable psychotic symptoms for ≥3 mo	N/A	Discontinuation of depot injections (n = 53)	N/A	1.5 y	Rating of 6 or 7 on any of the 3 BPRS psychotic symptom items	At 12 mo: 78% At 24 mo: 96% At 36 mo: 98%	N/A
Mayoral van Son et al. (2016)	Non-randomized, open-label intervention	First-episode non-affective psychosis (DSM-IV), n = 68	≥18 mo on maintenance treatment, ≥12 mo of clinical remission, ≥6 mo of functional recovery, and ≥3 mo stable at lowest effective doses	Discontinuation group: 106.8 (≤ 64.6) Maintenance group: 138.3 (≤ 80.7)	Gradual downward titration (n = 46)	Continuation of AP(s) originally taken prior to study entry (n = 22)	3 y	Rating of ≥5 on any key BPRS symptom items, CGI scale rating of ≥6 and a CGI change score of “much worse” or “very much worse”, hospitalization for psychotic psychopathology, or completed suicide	67.4%	31.8%
Reviews										
Gilbert et al. (1995)	Literature review of 66 studies	n = 4365	N/A	N/A	Refer to individual studies	Refer to individual studies	An average of 9.7 mo	Refer to individual studies	53%	16%
Viguera et al. (1997)	Review of 11 studies	n = 1210	Clinically stable status	N/A	Abrupt discontinuation (n = 1006) Gradual (tapering over at least 3 wk) discontinuation (n = 204)		Average of 54 ± 46 wk (range: 10 wk to 4 y)	Clearly worse clinically or by ratings; antipsychotic re-treatment required; hospitalization required	Abrupt discontinuation (within 10.2 ± 0.6 wk): 25% Abrupt discontinuation (within 30.3 ± 15.4 wk): 50% Gradual discontinuation (within 15.0 ± 1.0 wk): 25%	N/A
Thompson et al. (2018)	Systematic review of 7 studies	n = 520	Refer to individual studies	N/A	Refer to individual studies	Refer to individual studies	1 to 2 y	Refer to individual studies	53%	19%

Abbreviations: AP, antipsychotic; BPS, brief psychotic disorder; CGI, Clinical Global Impression; CPZ, chlorpromazine; DSM-IV, Diagnostic and Statistical Manual of Mental Diseases Fourth Edition; GAF, Global Assessment of Functioning; ICD, International Classification of Diseases; α-LA, alpha-lipoic acid; NOS, not otherwise specified; PANSS, Positive and Negative Syndrome Scale; PSE, Present State Examination; ω-3 PUFA, omega-3 polyunsaturated fatty acids; RCT, randomized controlled trial; RDC, Research Diagnostic Criteria; S, schizophrenia; SCA, schizoaffective; SCAN, Schedules for Clinical Assessment in Neuropsychiatry; SCID-IV, Structured Clinical Interview for DSM-IV; SCP, schizophreniform.

In a double-blind RCT by Kane et al. (1982), 28 patients in remission following FEP were randomized to receive either fluphenazine or placebo for 12 months, and relapses were seen in 7 out of 17 patients (41%) who received placebo and 0 of the 11 patients who received fluphenazine. The relatively low relapse rates in that study may be attributable to the loss of follow-up of patients in the placebo group and diverse study population that included patients with diagnoses other than non-affective psychosis. This study, despite its small sample size, demonstrated that patients in remission from FEP should remain on prophylactic treatment to prevent relapses.

Crow et al. (1986) demonstrated that 46% of FEP patients on maintenance antipsychotic treatment (any of 5 different agents) and 62% of patients on placebo had experienced a relapse by the end of their study participation (i.e., 2 years since hospital discharge, relapse, or loss to follow-up). Although there was a relatively high relapse rate in the placebo group, it is possible that the rate was still underestimated because of inclusion of any inpatients with a first-episode non-affective psychosis, irrespective of the diagnosis. In addition, the follow-up assessments were carried out at irregular time intervals, which may have introduced recall bias and led to inaccurate calculation of the number of recurrences. Despite these limitations, the medications were significantly effective for reducing the relapse rates. Both studies involved antipsychotic discontinuation relatively early (i.e., 3–4 months) after first psychotic episode (Kane et al., 1982; Crow et al., 1986).

Gilbert et al. (1995) reviewed 66 studies, including 4365 patients of the outcomes after neuroleptic withdrawal in patients with schizophrenia, and found a mean cumulative relapse rate of 53% in patients who discontinued their medications compared with 16% in patients who continued their medications over an average follow-up of 9.7 months. They highlighted it is important to note the variance in methodological aspects within and between the reviewed studies, particularly regarding the criteria for participants’ inclusion, relapse, as well as the duration and type of maintenance treatment provided. Similarly, a recent systematic review of 7 studies also found a pooled relapse rate of 53% for the medication discontinuation group and 19% for the maintenance group over a maximum follow-up of 2 years (Thompson et al., 2018).

Gitlin et al. (2001) then sought to perform a methodologically sound RCT to investigate the effects of a “targeted medication approach” on exacerbation/relapse rates. Patients with recent-onset schizophrenia who have received depot medication (fluphenazine decanoate) for at least 12 months were included in the 24-week double-blind cross-over trial. The patients were randomized to maintenance fluphenazine decanoate or placebo groups for 12 weeks followed by cross-over to the other treatment group for another 12 weeks. Participants who remained clinically stable at the end of the 24-week study were withdrawn from their treatment groups and followed-up for 18 months. During the open withdrawal phase of the study, 78% of participants relapsed in the first year and 98% relapsed in the second year. These high relapse rates warrant caution against early medication discontinuation. However, there were no significant differences in plasma drug levels between the maintenance therapy and placebo groups; therefore, there was no effective placebo control group. In addition, the drug levels in plasma before medication withdrawal or at relapse were not associated with the time to relapse. Nonetheless, with high relapse rates following medication discontinuation, the authors of this trial advised against early antipsychotic discontinuation.

### Long-Term Outcomes After Antipsychotic Discontinuation

Several studies have demonstrated the short-term risks of antipsychotic withdrawal (Kane et al., 1982; Crow et al., 1986; Gilbert et al., 1995; Gitlin et al., 2001; Thompson et al., 2018). Although the long-term outcomes are equally important, there is a paucity of long-term studies of outcomes of antipsychotic withdrawal. Such studies are complicated to perform because of the logistics required to recruit and randomize the patients as well as the high drop-out and non-compliance rates among patients with psychosis. Whereas short-term studies pinpoint the cautionary rates of relapse, 2 long-term follow-up studies were set to explore whether favorable outcomes would take place 7 or 10 years after a trial of antipsychotic discontinuation in remitted FEP patients (Wunderink et al., 2013; Hui et al., 2018) (Table 1).

Wunderink et al. (2007) conducted an open-label RCT, for 18 months, on 128 remitted FEP patients and demonstrated relapse rates of 43% and 21% in the dose-reduction/discontinuation and maintenance groups, respectively. Similarly, in a study by Chen et al. (2010), only 1 conducted in Asia, they performed a double-blinded RCT on 178 FEP patients free of symptoms for ≥1 year and demonstrated a relapse rate of 41% with quetiapine and 79% with placebo at the 12-month follow-up. The results of these RCTs agree with those of previous studies that patients have a higher risk for relapse with antipsychotic discontinuation than with maintenance treatment.

Researchers of the 2 previously mentioned studies performed follow-up of their respective trial patients. Wunderink et al. (2013) evaluated the rates of functional remission, symptomatic remission, and a combination of both for ≥6 months (i.e., recovery). The rates of symptomatic remission did not significantly differ between the dose-reduction/discontinuation and maintenance therapy groups after 7 years of trial. However, functional remission and recovery rates were significantly higher in the dose-reduction/discontinuation than in the maintenance groups. The high relapse rates in the dose-reduction/discontinuation group in the original trial had leveled off after 3 years of follow-up. Although Wunderink et al. (2013) concluded that early antipsychotic discontinuation was advantageous for FEP patients, only 20% of the patients in dose-reduction/discontinuation group (n = 13) were able to discontinue antipsychotics, while all other patients either never stopped antipsychotics or restarted them shortly after an attempt to discontinue (Wunderink et al., 2007).

Therefore, the results of small trials should be interpreted with caution. Hui et al. (2018) recently conducted a 10-year follow-up of the participants in the Chen et al. (2010) study. They found that at the 10-year follow-up, 21% and 39% of patients in the quetiapine and discontinuation groups, respectively, had poor long-term clinical outcomes (i.e., clozapine use, persistent positive symptoms, or suicide). Further analyses revealed that a recurring episode within the second or third year since onset mediates the association between antipsychotic discontinuation and poor long-term outcome. Rather than encouraging early dose reduction or discontinuation, Hui et al. (2018) present advisory data against it—that a minimum of 3 years of maintenance treatment should be followed through to prevent suboptimal clinical outcomes.

Short-term studies have demonstrated a high risk for relapse with antipsychotic discontinuation and have advised against the approach. However, long-term studies of antipsychotic discontinuation have had contradictory results. Wunderink et al. (2007, 2013) indicated potential benefits of ceasing medication early, while Chen et al. (2010) and Hui et al. (2018) found it to precede poorer clinical outcomes at 10 years. Therefore, whether early discontinuation or a minimum of 3 years on maintenance treatment as suggested by Hui et al. (2018) instigates optimal long-term outcomes is an issue of contention.

### Methodology and Quality Assessment

Many attempts have been made since the 1980s to evaluate the feasibility of antipsychotic discontinuation. Viguera et al. (1997) reviewed all the relevant studies on this topic and identified 2 key methodological flaws: variable antipsychotic doses (200–1150 and 175–890 chlorpromazine equivalent mg/d for abrupt and gradual discontinuation, respectively) and variable definitions of remission (although all studies reported that patients were “clinically stable”).

Better designed studies have been published since 2000. However, a careful review of these studies identifies several issues. First, Andreasen’s criteria were most frequently used to define remission. The Andreasen’s criteria define remission as a score ≤3 on each item of Positive and Negative Syndrome Scale (PANSS) and Brief Psychiatric Rating Scale along with a score ≤2 on each item of Scale for the Assessment of Positive Symptoms (SAPS) and Scale for the Assessment of Negative Symptoms for ≥6 months (Andreasen et al., 2005). Because a systematic review reported a mean relapse rate of 77% and 90% by 1 and 2 years, respectively, following antipsychotic discontinuation (Zipursky et al., 2014), using the criteria for full recovery rather than remission criteria would be more appropriate. In the survey, Korean psychiatrists favored the definition of remission as the complete absence of positive and negative symptoms or scores ≤2 on each item of the PANSS (Lee et al., 2020). Asian psychiatrists considered “absence of any relapse following first episode” as the most important criterion for antipsychotic discontinuation (Hui et al., 2019). In this respect, defining remission while allowing a score of ≤4 on the PANSS positive subscale, as did Wunderink et al. (2007), would appear to be too liberal. Moreover, only 1 study (Mayoral van Son et al., 2016) used clinical recovery as a criterion for antipsychotic discontinuation.

Second, there was marked variation in the mean antipsychotic doses before discontinuation: long-acting risperidone injection (consta) 25–50 mg, haloperidol 2.5–4 mg, olanzapine 7.1 mg, and risperidone 2 mg. These doses are in fact comparable with the minimum effective daily dose/olanzapine 1 mg equivalents reported by Leucht et al. (2014): aripiprazole 10 mg, asenapine 10 mg, clozapine 300 mg, haloperidol 4 mg, iloperidone 8 mg, lurasidone 40 mg, olanzapine 7.5 mg, paliperidone 3 mg, quetiapine 150 mg, risperidone 2 mg, sertindole 12 mg, and ziprasidone 40 mg (1.33 mg, 1.33 mg, 40 mg, .53 mg, 1.07 mg, 5.33 mg, 1 mg, .4 mg, 20 mg, .27 mg, 1.60 mg, and 5.33 mg risperidone, respectively). These doses, however, were studied for maintenance treatment, not for discontinuation. To prepare for discontinuation, doses should be reduced to levels lower than those mentioned above. In all studies, doses were tapered over 1 to 6 months. However, 30%–40% of relapses occurred in the first 3 months after antipsychotic discontinuation (Gitlin et al., 2001; Chen et al., 2010; Boonstra et al., 2011). It is possible that the early relapses were moderated by the duration of antipsychotic intake preceding discontinuation due to the medication build-up of supersensitive dopamine receptors (Samaha et al., 2007). Therefore, antipsychotics should be tapered more gradually and cautiously. Future RCTs should evaluate the risks of relapse after dose reduction or discontinuation of typical D2 blockers and partial D2 agonists to determine when and how fast the antipsychotics should be tapered (Murray et al., 2016).

Third, relapses can be classified into mild (i.e., PANSS positive subscale score ≥3 or change in Clinical Global Impression score ≥5 for ≥1 week), moderate, or severe (i.e., PANSS positive subscale score ≥5 or change in Clinical Global Impression score ≥6 for ≥1 week). If recovery is used as the criterion for antipsychotic discontinuation, it may be justified to use the criteria for mild relapses. As for the duration of re-emerging symptoms, which justifies the decision to restart antipsychotics, the 1-week criterion could be modified depending on the availability of proactive psychosocial support, 24-hour crisis intervention, or alternative medication (e.g., benzodiazepine) (Gaebel et al., 2016).

Finally, patients were regularly assessed for up to 3 years after antipsychotic discontinuation in most studies. However, only 1 study described what was included in these assessments (Emsley et al., 2014). Interestingly, in the Gaebel et al. (2016) study, patients were given the antipsychotic that the patient was already taking or the benzodiazepine lorazepam for early intervention. They chose these treatment options because a previous study had found no differences between these 2 drugs (Carpenter et al., 1999). Therefore, if the severity of re-emerging positive symptoms is mild, we can try using benzodiazepine first and use antipsychotics only when symptoms do not subside after more than 1 week.

Using the GRADE approach to assess the quality of RCTs (Table 2), we found serious or very serious problems in the study design and results. The study limitations included considering drop-out patients as having no relapse, unclear definition of remission, unclear allocation concealment, open-label study designs, diagnostic heterogeneity, and failure to report outcomes for the control group. Imprecision in these studies was evident by the small number of included patients, few relapses, wide confidence intervals, and confidence intervals including the value 1. Most studies were low or very low quality, except for 2 studies that were moderate quality (Chen et al., 2010; Hui et al., 2018). All studies, except for 1 (Wunderink et al., 2013), advised against antipsychotic discontinuation because of high relapse rates (19%–82% at a mean follow-up of 1 year).

**Table 2.** GRADE Evidence Profile: Studies on Antipsychotic Discontinuation

						Summary of findings						
	Quality assessment					No. of patients			Absolute risk			
Study	Limitations	Inconsistency	Indirectness	Imprecision	Publication bias	Discontinuation^*a*^	Maintenance	Relative risk (95% CI)	Control risk	Risk difference (95% CI)	Quality of evidence	Recommendation
Kane et al. (1982)	Very serious limitation^*b*^	No serious inconsistency	No serious indirectness	Very serious imprecision^*i*^	Undetected	7/17	0/11	Not estimable (no events)	0 per 1000	41.18% (17.78 to 64.57)	⊕⊕〇〇Low	No recommendation
Crow et al. (1986)	Very serious limitation^*b*^	No serious inconsistency	No serious indirectness	Very serious imprecision^*j*^	Undetected	41/66	25/54	1.34 (0.95 to 1.89)	463 per 1000	15.82% (−1.89 to 33.54)	⊕⊕〇〇Low	No recommendation
McCreadie et al. (1989)	Very serious limitation^*c*^	No serious inconsistency	No serious indirectness	Very serious imprecision^*i*^	Industry funded	4/7	0/8	Not estimable (no events)	0 per 1000	57.14% (20.49 to 93.8)	⊕⊕〇〇Low	No recommendation
Wunderink et al. (2007)	Very serious limitation^*d*^	No serious inconsistency	No serious indirectness	No serious imprecision	Industry funded partially	28/65	13/63	2.09 (1.19 to 3.65)	210 per 1000	22.44% (6.80 to 38.09)	⊕⊕〇〇Low	No recommendation
Chen et al. (2010)	Serious limitation^*e*^	No serious inconsistency	No serious indirectness	No serious imprecision	Industry funded partially	56/89	27/89	2.07 (1.46 to 2.95)	303 per 1000	32.6% (18.73 to 46.44)	⊕⊕⊕〇Moderate	No recommendation
Boonstra et al. (2011)	Serious limitation^*f*^	No serious inconsistency	No serious indirectness	Very serious imprecision^*j*^	Industry funded partially	10/11	4/9	2.05 (0.96 to 4.35)	444 per 1000	46.46% (9.83 to 83.1)	⊕⊕〇〇Low	No recommendation
Gaebel et al. (2011)	Very serious limitation^*g*^	No serious inconsistency	No serious indirectness	Very serious imprecision^*k*^	Undetected	4/21	0/23	Not estimable (no events)	0 per 1000	19.05% (2.25 to 35.84)	⊕⊕〇〇Low	No recommendation
Wunderink et al. (2013)	Very serious limitation^*d*^	No serious inconsistency	No serious indirectness	Serious imprecision^*l*^	Industry funded	21/52	9/51	2.29 (21.2 to 38.56)	176 per 1000	22.74% (5.79 to 39.69)	⊕⊕〇〇Low	Conditional
Emsley et al. (2014)	No serious limitation	No serious inconsistency	Serious indirectness (only 2 active comparators without control)	Very serious imprecision^*j*^	Undetected	9/12	19/21	0.83 (0.58 to 1.18)	No control	−15.48% (−43 to 12.05)	⊕⊕〇〇Low	No recommendation
Gaebel et al. (2016)	Very serious limitation^*h*^	No serious inconsistency	Serious indirectness (no results on control group)	Serious imprecision^*m*^	Undetected	10/19	Not available	Not estimable	Not available	Not estimable	⊕〇〇〇 Very low	No recommendation
Hui et al. (2018)	Serious limitation^*e*^	No serious inconsistency	No serious indirectness	No serious imprecision	Industry funded partially	35/89	19/89	1.84 (1.15–2.96)	210 per 1000	17.98% (4.73 to 31.22)	⊕⊕⊕〇Moderate	No recommendation

^*a*^Include also dose reduction or intermittent treatment.

^*b*^Drop out cases considered as non-relapse, poor definition of remission.

^*c*^Unclear allocation concealment, poor definition of remission.

^*d*^Open label, diagnostic heterogeneity, poor definition of remission.

^*e*^Diagnostic heterogeneity, poor definition of remission.

^*f*^Open label.

^*g*^Open label, drop out cases considered as non-relapse, poor definition of remission.

^*h*^Open label, poor definition of remission, failure to report outcome of control group.

^*i*^Few patients, few events and wide CI

^*j*^Few patients, and CI include 1.0.

^*k*^Few events and wide CI.

^*l*^Wide CI.

^*m*^Few patients.

Most patients in these studies (approximately 90%) had schizophrenia spectrum disorders (schizophrenia, schizoaffective disorder, and schizophreniform disorder), and only a small proportion (approximately 10%) had psychotic disorder not otherwise specified (NOS), delusional disorder, or brief psychotic disorder. Based on these findings, we conclude that the current evidence is against antipsychotic discontinuation in patients with remitted FES spectrum disorders. However, this conclusion should be taken in the context that most RCTs were of low-to-moderate quality based on the GRADE (Table 3).

**Table 3.** Overall GRADE Evidence Profile

						Summary of findings					
	Quality assessment					Relapse rate (%)					
Study	Limitations	Inconsistency	Indirectness	Imprecision	Publication bias	Discontinuation	Maintenance	Relative risk (95% CI)	Risk difference (95% CI)	Quality of evidence	Recommendation
	Very serious limitation	No serious inconsistency	No serious indirectness	Very serious imprecision	Undetected	53	19	__	0.26 (0.18 to 0.34)	⊕⊕〇〇Low	No recommendation

### Characteristics of Patients Who Discontinued Antipsychotics

It is important to identify patients who may safely discontinue antipsychotics after remission. The strongest predictor of relapse is medication non-compliance. However, relapse may also be predicted by certain psychosocial factors, including poor premorbid adjustment (Robinson et al., 1999; Larsen et al., 2000; Malla and Payne, 2005), long duration of untreated psychosis (Alvarez-Jimenez et al., 2012), expressed emotions in the household (Pourmand et al., 2005), and substance misuse (Barnett et al., 2007; Larsen et al., 2006). Decline in cognitive functions, including general memory (Verdoux and Liraud, 2000), set shifting (Chen et al., 2005), visual working memory (Hui et al., 2016), visual reproduction (Verdoux et al., 2000), verbal learning (Rund et al., 2007), attention, and processing speed (Wölwer et al., 2008), also predict relapse. However, these predictors were identified from naturalistic studies (where discontinuation is one of those predictors) that did not exclusively investigate the outcome of patients who discontinued antipsychotics. In addition, more pertinent to the issue of antipsychotic discontinuation is to identify factors that pinpoint patients who can do so without relapsing, evidence of which is currently limited.

Several studies have attempted to identify characteristics of patients with psychosis or schizophrenia who can discontinue antipsychotics without experiencing a relapse (Table 4). Fenton and Glashan (1987) retrospectively reviewed the data from 23 patients with chronic schizophrenia who remained stable despite not taking antipsychotics for an average of 15 years. They identified better premorbid occupational and social competence, fewer hebephrenic traits, and better regulation of depressed mood during the first hospital admission in these patients compared with other patients. Another research group identified a subgroup of first- or second-episode schizophrenia patients from the Soteria Project who received specialized psychosocial interventions and minimal or no medications during a 2-year follow-up period (Bola and Mosher, 2002). These patients had favorable outcomes in terms of hospitalization rates, independent living, psychopathology, and social and occupational functioning. In a quasi-experiment, Bola et al. (2006) compared the outcomes of patients who received family-oriented psychosocial interventions during a 3-week antipsychotic-free period with those of patients who received psychosocial interventions and antipsychotics. The 2-year study was completed by 46% of the participants without the need for antipsychotics. Different variables within the same domains of good prognosis and fewer schizophrenia symptoms predicted antipsychotic-free response or nonresponse with 74% accuracy.

**Table 4.** Key Results of Studies Investigating Characteristics of Patients Who Discontinued Antipsychotics

Study (design)	Diagnoses and total sample size	Definition of remission	Outcome of discontinuation	Characteristics associated with patients who were medication-free
Fenton and McGlashan (1987) (Retrospective study)	Chronic S; n = 23	Clinical global outcome score of moderate or better, never re-hospitalized, and no psychotropic medication use during follow-up period	Over average of 15 y: patients who discontinued AP sustained remission	Better psychosocial performance and acquired skills, more favorable premorbid occupational and social adjustments, fewer hebephrenic traits, and better preservation of depressed mood
Bola and Mosher (2002) (Retrospective exploratory study)	S (DSM-II); n = 179	No information	At 2-y follow-up: better outcomes on composite outcome scale (comprising of rehospitalization, psychopathology, independent living, social and occupational functioning) compared with those on AP continuously	Being older, higher score on Goldstein Adolescent Social Competence Scale, and fewer symptoms
Bola et al. (2006) (Quasi-experimental study)	Non-affective psychosis (DSMIII-R); n = 135	No information	At 2 y: 46.3% completed 2-y study without AP use	Higher score on GRIP on Life scale, parents had no mental health treatment history, higher scores on items “wahnstimmung” (sense of inner meaning or significance) and distorted speech of Comprehensive Psychopathology Rating Scale
Harrow and Jobe (2007) (Naturalistic observational study)	S (DSM-III); n = 64	(Definition of recovery) Absence of positive and negative symptoms throughout follow-up period and adequate psychosocial functioning; scoring 1 or 2 on LKP scale	At 10 y: 23% had “psychotic activity” At 15 y: 28% had “signs of psychotic activity” and 40% were in recovery	Better developmental achievements prior to onset, more favorable attitudinal approaches, more internal resources, and favorable prognostic factors
Harrow et al. (2012) (20-year longitudinal study)	S (DSM-III); n = 70	(Definition of recovery) Absence of positive or negative symptoms, no rehospitalizations during follow-up year and scores ≥2 on Strauss–Carpenter scales	At 4.5-, 7.5-, 10-, 15-, 20-y follow-up: less likely to present psychotic symptoms and experienced more periods of recovery	Greater resilience, more favorable premorbid developmental achievements, more internal resources, favorable prognostic factors and better neurocognitive functioning
Larsen-Barr et al. (2018) (Cross-sectional survey)	Psychosis, with a history of antipsychotic use; n = 144	No information	For varying periods of time: 55% successfully stopped all AP for some time	Patients who received support of various types were more likely to have successfully discontinued APs
Bowtell et al (2018b) (Review)	Varies between studies	Varies between studies	Proportion of individuals who experienced relapse following discontinuation ranged from 19.4% to 97% across 11 studies. No information regarding the remission rate of discontinuation group or comparing that with the maintained treatment group.	More years of education, better clinician-rated prognosis at baseline, and sustained remission at 2 y, were predictors of sustained remission following discontinuation. Several demographic and clinical factors found to be predictive of relapse. However, each predictive finding was reported only in 1 cohort of individuals such that no predictive findings were replicated across studies.
Tani et al. (2018) (Review)	Varies between studies	Varies between studies	Mean relapse rate after discontinuation: 38.3% per year	Older age, on a lower dose of AP prior to discontinuation, shorter DUP, older age at illness onset, better social functioning, less severe positive symptoms at baseline, and fewer previous relapses
Torgalsbøen et al. (2018) (Naturalistic observational study)	S (DSM-IV), n = 28	For ≥2 y, scores on items of PANSS must be ≤3; scores on BPRS also ≤3; item scores on SAPS and SANS ≤2 (additionally for full recovery) Working or studying, living independently, and at least 1 weekly social activity	No information	Fully recovered first-episode schizophrenia patients (where 10% of them no longer required antipsychotic medication) had better resilience
Fu et al. (2019) (Naturalistic observational study)	S (DSM-IV), n = 28	For ≥2 y, scores on items of PANSS must be ≤3; scores on BPRS also ≤3; item scores on SAPS and SANS ≤2 (additionally for full recovery) Working or studying, living independently, and at least 1 weekly social activity	Achieved recovery at earlier time point and experienced no relapses during follow-up periods	Use of active coping strategies such as being more aware of symptoms, mindful breathing, and changing of negative thought patterns promotes better recovery outcome. Larger improvements in processing speed and occupational functioning among patients not on antipsychotics than those who remained on antipsychotics.

Abbreviations: AP, antipsychotic; BPRS, Brief Psychiatric Rating Scale; DSM, Diagnostic and Statistical Manual of Mental Diseases; DUP, duration of untreated psychosis; LKP, Levenstein-Klein-Pollack scale; PANSS, Positive and Negative Syndrome Scale; S, schizophrenia; SANS, Scale for the Assessment of Negative Symptoms; SAPS, Scale for the Assessment of Positive Symptoms.

Using a different approach, Harrow and Jobe (2007) conducted a 15-year prospective longitudinal study to compare the premorbid and personality factors between schizophrenia patients on and off antipsychotics. They found that patients not on antipsychotics had longer periods of recovery and better functioning than those on antipsychotics. Patients who did not relapse after antipsychotic discontinuation had better premorbid functional development, more favorable personality traits, better prognostic factors, and greater resilience than patients who relapsed. Similarly, Torgalsbøen et al. (2018) identified resilience to be associated with fully recovered FES patients, with 10% of patients not on any treatment.

A secondary analysis performed using the protocol of Chen et al. (2010) evaluated the predictors of relapse for remitted FEP patients, both on and off antipsychotics (Hui et al., 2013). For patients who discontinued antipsychotics, relapse over the next year was predicted by a diagnosis of schizophrenia, poor semantic fluency, and higher blink rate.

In a longitudinal study by Harrow et al. (2012), 139 patients with psychosis from the Chicago Follow-up Study were followed-up 7 times over 20 years. Schizophrenia patients who remained stable without antipsychotics did not relapse more frequently than those who remained on maintenance antipsychotics. Greater resilience, better prognostic factors, premorbid functional development, less vulnerability towards anxiety, and better cognition were seen more frequently in patients off antipsychotics than on antipsychotics.

A recent cross-sectional survey by Larsen-Barr et al. (2018) observed that 105 out of 144 current or past antipsychotic users have attempted antipsychotic discontinuation at least once. In 55% of these attempts, patients received professional, service user, or peer support; this support had a positive association with successful attempts and negative association with relapses during withdrawal and need for antipsychotics. Hui et al. (2018) examined the 10-year clinical outcomes of the 178 FEP patients included in a previous trial (Chen et al., 2010) and evaluated the factors that predict successful antipsychotic discontinuation. Antipsychotics were successfully discontinued for 2 years, without any positive symptoms resurfacing, in 16% of FEP patients. Absence of relapses is, therefore, the most significant predictor of successful antipsychotic discontinuation. The other predictors include male sex, shorter duration of untreated psychosis, a diagnosis of non-schizophrenia spectrum disorder, better functioning at the initial phase of treatment, lower interference effect during the Stroop task, and remaining relapse-free while on placebo during RCT.

In another longitudinal study, Fu et al. (2019) identified 10 out of 28 FES patients who had recovered at the eighth follow-up during 6–8 years of follow-up. The processing speed and occupational functioning improved significantly in patients who were not on antipsychotics compared with those who remained on antipsychotics. Importantly, many patients used active coping strategies for recovery (e.g., changing negative thought patterns, improving understanding of symptoms, and practicing mindful breathing).

Tani et al. (2018) conducted a systematic review of 37 studies and observed that safe and successful antipsychotic discontinuation was predicted by lower antipsychotic dose prior to discontinuation, shorter duration of untreated psychosis, older age at disease onset, older age at present, better social functioning, milder positive symptoms at onset, and infrequent relapses. However, another review of studies conducted on FEP patients did not identify any replicated predictors of remission after antipsychotic discontinuation (Bowtell et al., 2018b). In patients who responded well to the initial antipsychotics and had sustained remission with maintenance treatment, longer duration of remission predicted lower risk for relapse following antipsychotic discontinuation (Hui et al., 2019a).

Further research is needed to identify factors that can identify patients who will and will not relapse following antipsychotic discontinuation. Although some of these factors have been identified, findings from the previous studies need to be confirmed in future studies and biological markers need to be identified to understand the mechanisms underlying relapses. It is important to identify patients who are likely to benefit from antipsychotic discontinuation. It is evident that not all patients with psychosis require life-long treatment (Jablensky and Sartorius, 2008; Harrow et al., 2012; Zipursky et al., 2014), and many patients discontinue antipsychotics without taking professional advice (Lacro et al., 2002; Zygmunt et al., 2002; Fleischhacker et al., 2003). It is therefore essential to determine the safest method of discontinuing antipsychotics.

### Review of Clinical Practice Guidelines

More than 20 clinical practice guidelines and algorithms have been proposed since 2000 for the treatment of schizophrenia (Table 5). A systematic review reported that 10 out of 11 guidelines and algorithms on the long-term antipsychotic use in the maintenance phase of schizophrenia, published between 2000 and 2011, advised against antipsychotic discontinuation within 5 years of treatment (Takeuchi et al., 2012). While exclusively for patients with FES, 6 of the 11 guidelines partially recommended antipsychotic discontinuation after 1 to 2 years of treatment. Intermittent or targeted antipsychotic use (i.e., stepwise drug discontinuation and early drug use in case of prodromal or early symptoms of relapse) is a potential alternative to antipsychotic discontinuation, and 9 guidelines and algorithms mentioned this approach, but none recommended it. For maintenance of remission, only 1 guideline suggested dose reduction (not lower than one-half of the effective dose for the acute phase) (Verma et al., 2011), while none of the others mentioned about or simply did not recommend dose reduction or lower dose therapy during this phase. Another systematic review conducted by Hui et al. (2019b) included 18 clinical practice guidelines for the treatment of maintenance phase of FES, and none of them recommended antipsychotic discontinuation.

**Table 5.** Guidelines and Algorithms of Antipsychotic Maintenance Treatment for Schizophrenia Published After 2000

			Discontinuation (within 5 y)		
Guideline/algorithm	Country	Year	First-episode schizophrenia	Multiple-episode schizophrenia	Intermittent or targeted strategy
Maudsley 13th^*a*^	UK	2018	Not mentioned	Not mentioned	Not recommended
CPA	Canada	2017	Partially recommended (after 1.5 y)	Not mentioned	Not mentioned
IPS	India	2017	Partially recommended (after 1–2 y)	Not recommended	Not mentioned
A-P region^*b*^	Asia-Pacific region	2016	Not mentioned	Not mentioned	Not mentioned
DHMA	Denmark	2016	Not mentioned	Not mentioned	Not mentioned
RANZCP	Australia and New Zealand	2016	Partially recommended (after 2–5 y)	Not mentioned	Not mentioned
NICE	UK	2014	Partially recommended	Not mentioned	Not recommended
HSSP	USA	2013	Not mentioned	Not mentioned	Not mentioned
Meta-guidelines	NA	2013	Partially recommended	Not mentioned	Not mentioned
SIGN	UK	2013	Partially recommended (after 1.5 y)	Not mentioned	Not mentioned
WFSBP	International	2013	Partially recommended (after 1 y)	Partially recommended	Not recommended
BAP^*b*^	UK	2011	Not mentioned	Not mentioned	Not recommended
Leucht	NA	2011	Partially recommended (after 1–2 y)	Not recommended	Not recommended
MOH	Singapore	2011	Not mentioned	Not mentioned	Not mentioned
PORT	USA	2009	Not mentioned	Not recommended	Not recommended
TMAP	USA	2008	Partially recommended (after 2 y)	Not mentioned	Not recommended
American Psychiatric Association (APA)	USA	2004	Not recommended	Not recommended	Not recommended
IPAP	International	2004	Partially recommended (after 1 y)	Not recommended	Not recommended
ECP	USA	2003	Not mentioned	Not mentioned	Not mentioned
MSC	USA	2002	Not mentioned	Not mentioned	Not mentioned

Modified from Shimomura et al. 2020.

^*a*^Maudsley 13th partially recommended discontinuation for schizophrenia in general, without specifying first- or multiple-episode.

^*b*^A-P region and BAP in general did not recommend discontinuation, without specifying first- or multiple-episode.

Shimomura et al. (2020) updated their previous systematic review of 11 guidelines and algorithms published after 2013 and added newly published guidelines/algorithms to the review. They suggested that in terms of considering antipsychotic discontinuation during maintenance phase, these newly published guidelines/algorithms seemed to be inclined to shift from “not recommended” to “partially recommended,” not only for FES (7 newly published guidelines endorsed, 4 not mentioned), but also for schizophrenia in general (also 7 newly published guidelines endorsed, 4 not mentioned) after a period of 1 to 5 years of treatment, possibly due to the recent reports on favorable long-term outcome in FEP: (1) the dose reduction/discontinuation patients experienced twice the recovery rate of the maintenance treatment patients at 7 years of follow-up (Wunderink et al., 2013), and (2) pooled prevalence of recovery among 9642 individuals with FEP was 38% (mean follow-up 7.2 years) (Lally et al., 2017). It is noteworthy that only the APA guideline (Lehman et al., 2004) did not recommend antipsychotic discontinuation in FES patients. Although intermittent or targeted antipsychotic use was not endorsed by the new guidelines and algorithms, antipsychotic dose reduction was recommended by 1, partially recommended by 5, and not mentioned by 5 out of 11 guidelines and algorithms published since 2013. In comparison, among the 9 guidelines published before 2012, 2 recommended, 3 partially recommended, 2 did not recommend, and 1 recommended a lower dose strategy. Therefore, the newer guidelines are more accepting of dose reduction/lower dose strategy during maintenance phase.

In addition to considering the stage of illness (first episode vs multiple episodes), long-term treatment strategy (maintenance vs intermittent treatment), and dosage for maintenance (the same as that used in acute phase vs lower dose strategy), more details should be considered for achieving appropriate and successful antipsychotic discontinuation. As seen in most guidelines, the minimum duration of stabilization under long-term antipsychotic treatment, usually recognized as a pre-requisite of discontinuation, was not uniformly defined, varying from 1, 1.5, 1 to 2, 2, or even up to 2 to 5 years (Shimomura et al., 2020). In general, the clinical treatment guidelines for FEP recommended the duration of maintenance treatment to be between 1 and 2 years (International Early Psychosis Association Writing Group, 2005; Early Psychosis Guidelines Working Group, 2010; Abidi et al., 2017; Remington et al., 2017), while a recent study has proposed that a minimum of 3 years promotes optimal long-term outcomes (Hui et al., 2018). The discontinuation strategy (discontinuation within a few weeks vs gradual tapering down antipsychotic dosage) as well as the tempo of gradual reduction (that is, the proportion of dose reduced at a time and the interval between dose reductions) is arbitrarily determined, mainly based on doctor’s clinical judgment or shared decision-making with the patients. Only 1 guideline specifically advised to “stop gradually over at least 3–6 months with close follow-up” (Galletly et al., 2016). This variation in strategy based on clinician judgment may affect the outcomes after drug discontinuation (National collaborating centre for mental health, 2014) or dose reduction but has not received adequate attention (Liu et al., 2021). Moreover, only 2 guidelines suggested monitoring 2 years after discontinuation (Grover et al., 2017, National collaborating centre for mental health, 2014). Gaebel et al. (2019) reported that the clinical course following antipsychotic discontinuation is heterogeneous and complex. There was wide variation in the clinical outcomes of patients, including no relapses, acute or insidious relapses, or clinical deterioration following drug discontinuation. Nonetheless, there was a minority of patients who remained clinically stable after antipsychotic discontinuation, but no individual-level variables predicted successful antipsychotic discontinuation or higher risk for relapse following discontinuation (Bowtell et al., 2018a, 2018b).

Correll et al. (2018) critically appraised the risk-benefit ratio of long-term antipsychotic use in patients with schizophrenia and identified a small group of patients with no relapse despite prolonged periods of no antipsychotic use. However, they concluded that the evidence favored continuous antipsychotic use. In response to that study, several prominent researchers advised to “encourage a sense of curiosity about the possibility of dose reduction and discontinuation in appropriate patients (Marder and Zito, 2018),” to have “guidelines to be developed on when and how slowly to reduce antipsychotics, and in whom it is appropriate to eventually stop them (Murray and Di Forti, 2018),” and if guidelines are to be changed, “due to the subjectivity of psychiatric outcomes—there is room for interpretation, in the future the evidence will have to be presented such that patients can decide themselves” (Leucht, 2018).

### Ongoing Discontinuation Trials

Four prominent studies of medication discontinuation are underway (Table 6). The participants of these studies have been or will be randomized into maintenance or dose reduction strategy groups. If the dose is successfully reduced, participants can discontinue their antipsychotics completely. The primary outcomes of interest in these studies are the functional outcomes.

**Table 6.** Ongoing Studies Concerning Discontinuation of Antipsychotic Medication

Research	Country	Study design	Diagnoses	Total sample size	Age	Remission criteria	Remission duration	Method of AP discontinuation	Follow-up duration after discontinuation	Primary outcome	Primary outcome measures
TAILOR (Sturup et al., 2017)	Denmark	Open-label RCT; comparison of maintenance therapy and tapering/discontinuation with antipsychotic medication	First episode of schizophrenia or persistent delusional disorder (ICD10)	250	18 y old or older	All global scores in SAPS <3	Minimum 3 mo	Approximately 25% monthly reduction of initial dose. Next step dose maintained in 3-mo stabilization phase before final step of 3 mo tapering to discontinuation. If initial dose below minimum effective dose, first step is skipped.	5 y	Remission of psychotic symptoms and no antipsychotic medication after 1 y	SAPS
HAMLETT (Begemann et al., 2020)	Dutch	Single-blind RCT; comparison of maintenance therapy and tapering/discontinuation with antipsychotic medication	First episode of schizophrenia, schizoaffective disorder, schizophreniform disorder, brief psychotic disorder, delusional disorder, substance/medication-induced psychotic disorder, or those classified as unspecified schizophrenia spectrum and other psychotic disorders (DSM-5, or ICD10)	512	16 to 55 y old	≤3 on positive PANSS items	3–6 mo	Treating physicians prescribe tapering schedule that fits patient’s type and dose of baseline medication. Average duration until complete discontinuation is 3 mo.	4 y	Personal and social functioning	WHO-DAS 2.0 disability scale
REDUCE trial (Weller et al., 2019)	Australia	Single-blind RCT; comparison of antipsychotic dose reduction strategy with EBIRT group (DRS+) and antipsychotic maintenance treatment with EBIRT group (AMTx+)	First episode of a psychotic disorder or mood disorder with psychotic features (DSM 5)	180	15 to 25 y old	≤3 on hallucinations, unusual thought disorder, conceptual disorganization, and suspiciousness subscale items of BPRS for past 2 wk and past 3 mo based on systematic clinical file review and collateral information collected from participant’s treating team	Minimum 3 mo	Rate of tapering is 25% dose reduction (or as near to 25% as medication allows) of pre-reduction dose every month for 3 mo until participant reduces dose considered clinically safe	2 y	Social functioning	SOFAS
RADAR (Moncrieff et al., 2019)	UK	Open-label RCT; comparison of continuation of antipsychotic medication and gradual dose reduction until eventual discontinuation of antipsychotics	Clinical and/or ICD10 diagnosis of schizophrenia, schizoaffective disorder, delusional disorder or other non-affective psychosis; people with >1 episode of psychosis or schizophrenia	402	18 y old or older	None	None	Dose reduced incrementally every 1 or 2 mo. Most schedules aim for discontinuation within 12 mo. Participants are offered option to discontinue antipsychotic medication completely if reduction progresses well or to reduce to a very low dose, defined as equivalent of 2 mg/d haloperidol or less.	2 y	Social functioning	SFS

Abbreviations: DSM, Diagnostic and Statistical Manual of Mental Diseases; EBIRT, evidence-based intensive recovery treatment; ICD, International Classification of Diseases; SAPS, Scale for the Assessment of Positive Symptoms; SFS, Social Functioning Scale; SOFAS, Social and Occupational Functioning Scale.

#### Tailor

The TAILOR trial is being conducted in Denmark. Two hundred and fifty patients with schizophrenia (F20, except F20.6) or persistent delusional disorder (F22), in accordance with the International Classification of Disease 10th edition, will be recruited from the intensive early intervention program (OPUS) (Sturup et al., 2017). The study participants will have remission of psychotic symptoms for at least 3 months on antipsychotics (except clozapine). Remission will be defined as SAPS (all global scores <3). The study participants will be randomized into maintenance therapy or antipsychotic tapering/discontinuation groups for 1 year. The dose will be reduced by 25% of initial dose each month for 3 months and maintained for 3 months, followed by tapering of antipsychotic dose until discontinuation over 3 months. The last antipsychotic dose before discontinuation will be 25% of initial dose. If the initial dose was below the minimum effective dose (Leucht et al., 2014), the first step will be skipped. The tapering/discontinuation group will use a smartphone application to monitor early warning signs of relapse. The participants will be assessed at baseline and at 1-, 2-, and 5-year follow-up for positive and negative symptoms, side effects of antipsychotics, social function, cognitive function, perceived health status, patient satisfaction, substance and alcohol use, sexual function, and quality of life. The primary outcomes will be remission of psychosis (SAPS global scores ≤2 for ≥3 months) and no need for antipsychotics after 1 year.

#### Reduce Trial

The Reduce trial, being conducted in Australia, will include 180 patients with FEP from a specialist early-psychosis treatment setting (Weller et al., 2019). The participants of this trial scored ≤3 on the hallucinations, unusual thought disorder, conceptual disorganization, and suspiciousness items of the Brief Psychiatric Rating Scale (Ventura et al., 1993) for the past 2 weeks and 3 months based on the file review and information from the treating team, respectively. Participants will be randomized to dose reduction strategy with an evidence-based intensive recovery treatment (EBIRT) group (DRS+) or maintenance treatment with EBIRT group (AMTx+). Antipsychotics will be tapered under close medical supervision over 3 months after allocation to the DRS+ group to minimize the risk for relapse due to abrupt discontinuation. The dose will be reduced by 25% (or as close to 25% as the medication allows) of the pre-reduction dose every month for 3 months, until the participant is on a dose considered clinically safe, followed by antipsychotic discontinuation in some patients. There may be some variation in the dose reduction schedule. This trial will compare the effects of DRS+ and AMTx+ groups on vocational and social recovery, physical health, cognition, and brain volume. The primary aim of this study will be to compare the functional outcomes among young FEP patients in the DRS+ and AMTx+ groups during follow-up for 24 months. In addition, this study will determine the effects of antipsychotic maintenance treatment on brain volume changes in this population.

#### Handling Antipsychotic Medication Long-Term Evaluation of Targeted Treatment Study

The Handling Antipsychotic Medication Long-term Evaluation of Targeted Treatment study is underway in the Netherlands (Begemann et al., 2020). This trial will include 512 patients with symptomatic remission from FEP for 3–6 months. Participants with severe or life-threatening psychosis, self-harm, or violence will be excluded from the study. Remission was defined as scores ≤3 on positive PANSS items. FEP patients may have schizophrenia, schizoaffective disorder, schizophreniform disorder, brief psychotic disorder, delusional disorder, substance/medication-induced psychotic disorder, or unspecified schizophrenia spectrum and other psychotic disorders (Diagnostic and Statistical Manual of Mental Disorders-5 or International Classification of Diseases 10th edition). The participants will be randomized to either continue antipsychotics for at least 1 year after remission (maximum reduction of 25% of initial dose, or other antipsychotics at a similar dose range) or gradual dose reduction followed by antipsychotic discontinuation (Table 7). A plan of action describing early warning signs will be developed by the treating physician prior to tapering off medications. Patients will be able to find early warning signs (e.g., social withdrawal and sleep disturbances) in a booklet provided at the beginning of the study, and early warning signs will be noted by the patient, caregivers, and relatives of the patient. If signs of relapse occur, the antipsychotic doses will be increased. Patients were assessed at baseline, at 3 and 6 months post-baseline, and yearly during a follow-up period of 4 years. The primary outcome will be personal and social functioning, evaluated using the World Health Organization Disability Assessment Schedule 2.0.

**Table 7.** Tapering off Schedules for the HAMLETT

	Max doses	Tablets	Start	2 wk	4 wk	6 wk	8 wk	10 wk	12 wk	14 wk	16 wk	18 wk	20 wk	22 wk	24 wk	26 wk	28 wk	HS
Risperidone	10	0,5, 1, 2, 3, 4, 6	10	9	8	7	6	5	4	3	2	1,5	1	0,5	0,25	0.25	Stop	Stop
Olanzapine	20	2,5, 5, 10, 15, 20	20	17.5	17.5	15	12.5	10	7.5	5	5	2.5	2.5	1,25	1,25	Stop	Stop	Stop
Quetiapine	800	25, 100, 200, 300	800	700	600	500	400	300	250	200	150	100	75	50	25	25	Stop	Stop
Aripiprazole	20	5, 10, 15	20	15	15	12,5	12,5	10	10	7,5	7,5	5	5	2,5	2,5	Stop	Stop	Stop
Haloperidol	20	1, 5	20	18	16	14	13	12	10	8	6	4	3	2	1	1	Stop	Stop
Zuclopenthixol	40	2, 10, 25	40	36	32	28	24	20	18	16	12	10	8	4	2	2	Stop	Stop
Sulpiride	800	400, 50	800	600	500	450	400	350	300	250	200	150	100	50	50	Stop	Stop	Stop
Paliperidone	12	3, 6, 9	12	10,5	10,5	9	7,5	7,5	6	6	4,5	3	3	1,5	1,5	Stop	Stop	Stop
Pimozide	20	1, 4	20	18	16	14	12	10	8	7	6	4	3	2	1	1	Stop	Stop
Lurasidone	148	37, 74	148	111	111	92,5	92,5	74	74	55,5	55,5	37	37	18,5	18,5	Stop	Stop	Stop
Clozapine	900	25, 100, 200	900	700	600	500	400	350	300	250	200	150	100	50	25	25	Stop	Stop
Amisulpride	800	100, 400	800	700	600	500	400	350	300	250	200	150	100	50	50	Stop	Stop	Stop
Tiapride	600	100	600	500	450	400	350	300	250	200	150	100	100	50	50	Stop	Stop	Stop
Sertindole	24	4, 16	24	22	20	18	16	14	12	10	8	6	4	2	2	Stop	Stop	Stop
Chlor-protixene	300	15, 50	300	250	215	200	165	150	130	115	100	65	50	30	30	15	15	Stop

Permitted from principal investigator of the HAMLETT.

#### Research Into Antipsychotic Discontinuation and Reduction Trial

The Research into Antipsychotic Discontinuation and Reduction trial is being conducted in the United Kingdom (Moncrieff et al., 2019). This trial recruits patients with more than 1 episode of psychosis or schizophrenia, although other trials have recruited patients who had FEP. Following informed consent, patients will be randomized to receive either maintenance antipsychotics (continuing the current regime) or antipsychotic reduction strategy with gradual reduction in antipsychotic dose with the support of a psychiatrist. The dose will be reduced every 1–2 months, aiming to discontinue antipsychotics within 12 months. Participants who wish to discontinue antipsychotics will be supported, but those who wish to continue the antipsychotics will have their doses reduced to a very low level (i.e., equivalent to 2 mg/d haloperidol). The time required for antipsychotic tapering varies between different individuals, but the investigators estimate that it will take 6–18 months. The main outcome for this study will be the level of social functioning, and the patients will be followed-up for up to 2 years. The investigators will also evaluate the relapses, symptoms, medication side effects, employment status, and costs.

### Guidelines for Antipsychotic Discontinuation After First Episode of Schizophrenia Spectrum Disorders

The GRADE approach was used for evaluating the quality of evidence (Tables 2–3). Antipsychotic discontinuation could not be recommended in FEP or FES patients, albeit there was low quality of evidence. It is noteworthy that 18%–81% of patients remain relapse free at 9 months or 1 year follow-up. Furthermore, if we apply more strict criteria for the condition of discontinuation such as full recovery criteria and analyze outcome in more mild cases such as psychotic disorder NOS, the proportion of relapse-free patients would be much higher. It is of paramount importance to conduct more research using the strict criteria of full recovery and more detailed guidelines as to when and how antipsychotics can be tapered and discontinued. This would give us milestones to find characteristics of patients who can stop taking medication successfully and biomarkers in predicting safe discontinuation. The summarized guidelines below should be taken as those for future research based on the review of related literature and expert consensus and possibly for clinical practice with more careful attention. In addition, given that most of the participants were patients with FES spectrum disorders, the guideline is intended for those patients:

1. Antipsychotics should be continued for at least 1–3 years after the initial psychotic episode. In patients who wish to discontinue antipsychotics, the decision should be made after discussing the risks and benefits of discontinuation with the patients through a process of shared decision-making, empowering them to take ownership of their disease and recognizing their unique signatures and early warning signs for relapse. Patients should be informed of the relatively high risk for relapse and its sequelae and should be mentally prepared to manage a relapse. Patients should be warned that relapses can occur rapidly and may be associated with loss of insight and compromised help-seeking behavior.
2. Successful antipsychotic discontinuation is predicted by diagnosis other than schizophrenia, better premorbid social and occupational functioning, good social support, shorter duration of untreated psychosis, no cognitive impairment, favorable personality traits, greater resilience, and absence of suicidality or risky behaviors. Patients with a history of relapse after the index first episode should be advised against discontinuation. These predictors should be considered while making a decision regarding antipsychotic discontinuation.
3. Patients should have achieved symptomatic (score ≤2 on P1–3, N1, N4, N6, G5, and G9 of PANSS) and functional recovery for 6–12 months before discontinuing antipsychotics.
4. Drugs should be tapered gradually over 6–12 months with careful monitoring for re-emerging (attenuated) psychotic symptoms or any relapse signs. Doses should be reduced gradually, at an individualized rate, and each reduction should not exceed 25% of the previous dose. Final antipsychotic dose before discontinuation should be equivalent ≤1 mg risperidone. In the case of long-acting injectable antipsychotics, the lowest dose should be tried (25 mg for paliperidone palmitate), with regular or prolonged intervals between injections. For monthly aripiprazole injections, patients should be switched to oral antipsychotics, given that the lowest dose of aripiprazole once-monthly (300 mg) is comparable with an oral dose of 15 mg. Aripiprazole, a dopamine partial agonist, may be considered as a last step and safe antipsychotic drug in terms of minimizing dopamine super-sensitivity psychosis. With this ultra-low or lowest possible dose, the recovered state should be maintained for at least 3–6 months before discontinuation.
5. Dose reduction should be slowed or stopped in cases of early signs of relapse. After stabilization of the relapse symptoms, if a patient meets the criteria for full recovery, a much slower rate of dose reduction may be used to taper antipsychotics. If dose reduction fails, the previously used dose, which would be the lowest effective dose, should be maintained.
6. Psychosocial interventions aimed at improving self-efficacy, illness management, and social and occupational functioning should be implemented during the process of antipsychotic discontinuation. Family-oriented psychosocial interventions, aimed at educating and empowering caregivers in relapse prevention, should be implemented. Case management and ongoing support and monitoring should be continued for at least 2 years after antipsychotic discontinuation.
7. If attenuated positive symptoms emerge after antipsychotic discontinuation, intensive and frequent psychosocial interventions should be provided. The emerging symptoms should be assessed, and a decision should be taken to restart antipsychotics. Accurate assessment of emerging symptoms and careful decision to restarting antipsychotics should be performed. If the severity of re-emerging positive symptoms is mild and does not cause risky behaviors, consider using other medications such as benzodiazepine or antidepressant for a week. However, if symptoms do not subside or they worsen, the decision to restart antipsychotic medication should be made promptly through a process of shared decision making.

## Discussion

Antipsychotic discontinuation has been a long-standing clinical and medicolegal issue. On one hand, antipsychotic use is associated with obvious inconveniences, side effects, and self- and public stigmatization. For these reasons, up to 30% of FEP patients discontinue their antipsychotics within the first 9 months of treatment (Miller et al., 2011). On the other hand, accumulated data indicate that there is a high risk for relapse in patients with psychosis following antipsychotic discontinuation. Each relapse of psychosis is devastating and leads to irreversible damage; therefore, the high relapse risk cautions against early antipsychotic discontinuation. However, up to 10% of remitted FEP patients do not require life-long antipsychotics to maintain remission (Zipursky et al., 2014). The decision of antipsychotic (dis)continuation therefore presents a paradox for patients as much as it does for their clinicians—a relapse “precipitated” by ceasing medication could hold clinicians accountable, as do patients’ unnecessary suffering due to prolonged pharmacological treatment. The pivotal question of when and how antipsychotic discontinuation could be attempted remains unanswered. Such a decision about maintenance treatment involves a risk-benefit analysis on the detrimental side effects associated with continued medication and the risk of relapse associated with discontinuation.

Based on the best evidence and expert opinions, we developed these guidelines for antipsychotic discontinuation in patients with FES spectrum disorders. These guidelines will guide patients and clinicians on the safest approach to discontinue antipsychotics. The most crucial aspect of the guidelines was to adopt a more conservative stance about the criteria for discontinuation, that is, comprehensive recovery (absence of positive and negative symptoms and functional recovery) rather than only symptomatic remission. This is based on high relapse rates in previous studies after antipsychotic discontinuation and a conservative attitude of Asian psychiatrists. Even when successful antipsychotic discontinuation or dose reduction is not possible because of aggravating or reemerging symptoms or relapse, we believe that this would give us a golden opportunity to enhance a patient’s insight and subsequent medication adherence. Equally important is that by using the guidelines, we hope we can help identify the patients who do not need continuous medication. Balancing these 2 purposes of the guidelines would certainly lead to optimal outcomes of patients with FEP. Finally, we hope that studies will be conducted on antipsychotic discontinuation in Asian countries in the near future.

## Interest Statement

Y.C. Chung received chair honorarium from Otsuka. E.Y.H. Chen received speaker honoraria from Otsuka and DSK BioPharma, research funding from Otsuka, funding to attend conferences from Otsuka and DSK BioPharma, and participated in paid advisory boards for Jansen and DSK BioPharma. H. Takeuchi has research grants from Daiichi Sankyo and Novartis Pharma and received speaker’s fees from EA Pharma, Kyowa, Janssen, Lundbeck, Meiji Seika Pharma, Mitsubishi Tanabe Pharma, Mochida, Otsuka, Sumitomo Dainippon Pharma, Takeda, and Yoshitomiyakuhin. All other authors declare no competing interests.
